# Disturbance of hibernating bats due to researchers entering caves to conduct hibernacula surveys

**DOI:** 10.1038/s41598-024-64172-8

**Published:** 2024-06-12

**Authors:** Jericho C. Whiting, Bill Doering, Ken Aho, Bryan F. Bybee

**Affiliations:** 1 Department of Biology, Brigham Young University-Idaho, Rexburg, ID USA; 2 POWER Engineers, Inc., Meridian, ID USA; 3 Department of Biological Sciences, Idaho State University, Pocatello, ID USA; 4 Idaho National Laboratory, Idaho Falls, ID USA

**Keywords:** Acoustic sampling, *Corynorhinus townsendii*, Hibernacula, Hibernacula surveys, *Myotis ciliolabrum*, Animal behaviour, Conservation biology, Ecological modelling

## Abstract

Estimating population changes of bats is important for their conservation. Population estimates of hibernating bats are often calculated by researchers entering hibernacula to count bats; however, the disturbance caused by these surveys can cause bats to arouse unnaturally, fly, and lose body mass. We conducted 17 hibernacula surveys in 9 caves from 2013 to 2018 and used acoustic detectors to document cave-exiting bats the night following our surveys. We predicted that cave-exiting flights (i.e., bats flying out and then back into caves) of Townsend’s big-eared bats (*Corynorhinus townsendii*) and western small-footed myotis (*Myotis ciliolabrum*) would be higher the night following hibernacula surveys than on nights following no surveys. Those two species, however, did not fly out of caves more than predicted the night following 82% of surveys. Nonetheless, the activity of bats flying out of caves following surveys was related to a disturbance factor (i.e., number of researchers × total time in a cave). We produced a parsimonious model for predicting the probability of Townsend’s big-eared bats flying out of caves as a function of disturbance factor and ambient temperature. That model can be used to help biologists plan for the number of researchers, and the length of time those individuals are in a cave to minimize disturbing bats.

## Introduction

Caves are used for hibernation by 40% of bat species of the world, making them a limited and important resource for bat conservation^1–4^. Bats can use the same caves for decades^5–7^, because these features are permanent, thermally stable, and provide protection from climatic extremes^5,8,9^. Therefore caves are important for bat conservation, because these features are discrete resources that can be protected^10–12^, and biologists often know when and generally how long large concentrations of bats will occupy caves in winter^7,13^.

Many bat species in temperate environments hibernate in caves. During hibernation bats rely on stored energy when temperatures and insect abundance decrease^14–16^. Hibernation allows bats to persist for long periods of time while using little energy^9,17,18^. Duration of hibernation is generally determined by the length of time that bats cannot successfully forage^9,19,20^. Hibernating bats enter torpor (i.e., controlled reductions in body temperature and metabolism to conserve energy), but often arouse from torpor and fly in winter^17,21,22^. Arousal is due to internal cues or environmental changes^21,23,24^. Some bats arouse in winter to eat, drink, change positions or fly to another hibernaculum^1,25^. Large numbers of bats can congregate in caves during hibernation^5,6,26^, which can result in a high potential for bats to be disturbed when humans enter caves^2,5,27^.

Estimating long-term population changes of bats is important for targeting conservation efforts^28–31^. Often, population estimates are calculated by researchers entering caves to count bats during hibernation^26,32,33^, or to assess bat mortality from white-nose syndrome. Population surveys are one of the best methods to monitor changes over time, because bats return to the same hibernation sites annually^7,30,34^. Humans entering caves to conduct surveys can disturb bats during torpor due to the presence of lights and noise^5,35–39^. These disturbances can lead to elevated body temperatures, altered patterns of arousal, and/or a loss in body mass, leading to decreased survival in hibernating populations^36,38,40^. Population growth can be further affected by decreased annual reproductive rates of bats^1,5,41^.

Passive acoustic recorders can be used to quantify animal behavior^22^ and how human disturbance may affect behavior patterns^42,43^. Previously, researchers entering eight artificial bunkers and one mine during one winter to count bats caused some of those mammals to arouse from torpor and fly during hibernation^33,38^. Little is known, however, about how bats may react to researchers conducting hibernacula surveys in multiple caves across several years. We used passive acoustic detectors set at 9 cave openings to document cave-exiting flights of bats (i.e., bats flying out of caves and back into caves) before and after we conducted 17 hibernacula surveys from 2013 to 2018. We hypothesized that researchers entering caves to conduct hibernacula surveys would increase cave-exiting flights of Townsend’s big-eared bats (*Corynorhinus townsendii*) and western small-footed myotis (*Myotis ciliolabrum*) within the 24 h following surveys, as documented in other studies^33,38^. Specifically, we predicted that the number of acoustic call files recorded the night following hibernacula surveys would be larger than the number of acoustic call files recorded during nights following no surveys. Our results provide further information on disturbance to bats caused by researchers entering caves, which can be used to plan surveys with minimal effects to hibernating bat populations.

## Study area

We surveyed hibernacula and recorded cave-exiting flight of Townsend’s big-eared bats and western small-footed myotis in 9 caves in Idaho, USA, on the Idaho National Laboratory Site (INL Site; 43°36.015N, 112°51.441W). Those species comprised > 99% of bats observed historically during hibernacula surveys^6,44^ and also were the most recorded species acoustically during hibernation in our study area^22^. The INL Site has been closed to public access since the 1940s^45^. That area was patrolled by security guards to limit unauthorized access; therefore, we assumed illegal entry into caves was negligible during our study. The INL Site ecosystem is a sagebrush (*Artemisia tridentata*)-steppe desert^7,22^ with hot, dry summers and cold winters^44,45^.

Caves used by hibernating bats in our study area were lava blisters and tubes, and none were gated^6^. The mean distance from a cave to all other caves in our study area was 15 km (s.d. = 4.6 km)^22^. The maximum distance travelled between hibernacula across years by 101 banded Townsend’s big-eared bats in our study area was 8.3 km^46^. We do not provide cave names and locations to protect those resources^2^, however, we assigned letter and number combinations to caves that corresponded with cave designations in Whiting et al.^6^.

## Methods

### Hibernacula surveys

We conducted one yearly survey per cave during December–March in 2013, 2014, 2015, and 2018 (Table 1). Surveys were conducted between 9:38 a.m. and 4:16 p.m. The mean elapsed time between the end of our surveys and the beginning of sunset was 4 h and 55 min (s.d. = 2 h), at which time bats would start flying out of caves naturally^22^. That difference left sufficient time for bats to be detected by our acoustic equipment if they had been disturbed by us conducting surveys, as bats continue flying up to 10 h after arousal from human disturbance^33,38^. We conducted hibernacula surveys with at least 3 surveyors for safety (mean = 3, s.d. = 0.5, range = 3 to 4). We conducted surveys in a similar manner each year by walking the length of the cave and searching for bats with 90–130 lm power headlamps and 210 lm power hand-held flashlights, and communication was minimal^38^. We did not touch any bats during surveys. We performed all surveys in accordance to established protocols to minimize disturbance of hibernating bats^2,47^. Entering caves to count hibernating bats was approved by the Idaho National Laboratory Site Cave Protection and Access Committee (permit number OS-ESD-16–108).

**Table 1:** Cave number and length, survey date, number of Townsend’s big-eared bats (COTO), western small-footed myotis (MYCI), and researchers that entered caves to conduct surveys, as well as the amount of time researchers spent conducting surveys and a derived researcher disturbance factor (i.e., number of researchers × total time in a cave in min.) for hibernacula surveys conducted in southeastern Idaho.

Cave	Length (m)	Survey date	COTO	MYCI	# of researchers	Total survey time (min.)	Disturbance factor
C2	288	2/15/2013	112	0	4	79	316
		2/13/2018	66	0	4	118	472
C14	25	2/11/2013	3	0	3	19	57
		2/11/2015	2	0	3	25	75
C19	250	2/5/2013	23	2	3	115	345
		2/27/2015	25	0	3	74	222
C36	117	2/8/2013	5	0	3	69	207
C40	615	2/11/2013	391	9	3	127	381
		3/20/2015^b^	443	9	4	133	532
		1/30/2018^a,b^	373	23	4	175	700
C41	148	2/14/2013	1	0	3	48	144
		2/11/2015	2	0	3	52	156
C46	123	2/5/2013	3	0	3	59	177
		2/11/2015	3	0	3	30	90
C47	65	2/25/2013	25	0	3	37	111
C54	311	2/25/2013	130	24	3	106	318
		12/22/2014^b^	127	6	4	75	300

^a^Cave and year that western small-footed myotis exited a cave the night following our survey.

^b^Cave and year that Townsend’s big-eared bats exited caves the night following our survey.

### Passive acoustic monitoring

We set 9 Anabat SDI and SDII detectors (Titley Scientific, Columbia, MO)—one at each cave—within a mean of 3 m (s.d. = 2.5 m) of the outside of cave openings during hibernation (November to March)^22,48^. We sent detectors and microphones to Titley Scientific approximately every two years to be tested and serviced^22,48^, and we moved detectors across years among caves throughout the study based on servicing schedules and field logistics^22^. Detectors were powered by external batteries and solar panels^17,21,22^. Each directional microphone was equipped with a BatHat to reduce damage to equipment from inclement weather^48,49^. Because of cave characteristics, all but one cave (C54) had reflector plates oriented at 45° angle from the center axis of the microphone^49^. The lack of a reflector plate at cave C54 did not reduce the number of calls received for Townsend’s big-eared bats or western small-footed myotis compared with other caves^22^. Detectors recorded at least from sunset to sunrise^22^. The division ratio was set at eight^50^, and we also adjusted the sensitivity to exclude ambient noise^51^.

Microphones were about 3 m above the ground and positioned so the center axis of the zone of reception was approximately 15° above the horizon^22^. We oriented microphones to maximize detection of bats flying past the dripline of the cave while trying to avoid recording noise and echoes^22,48^. When triggered by bats flying outside of hibernacula, detectors created one, ≤ 15 s. call file, labeled with a date and time stamp^22,48^. Bats flying out of a cave after conducting hibernacula surveys were not a measure of a population response to researchers entering a cave, because one bat that aroused could have flown in front of the detector multiple times; therefore, our acoustic detectors measured the total cave-exiting bat activity, representative of only a portion of the population^38^. We used AnaLookW to filter files for bat search-phase calls (i.e., files containing ≥ 1 search-phase echolocation sequence of ≥ 2 echolocation pulses) by call structure by species^22,52,53^, and one coauthor (B. Doering) manually vetted all files that passed filters^22,48^. Our detector array has been successful at documenting extensive, natural cave-exiting flight of Townsend’s big-eared bats and western small-footed myotis in our study area^22,48,54^.

### Statistical analyses

**Figure 1:**
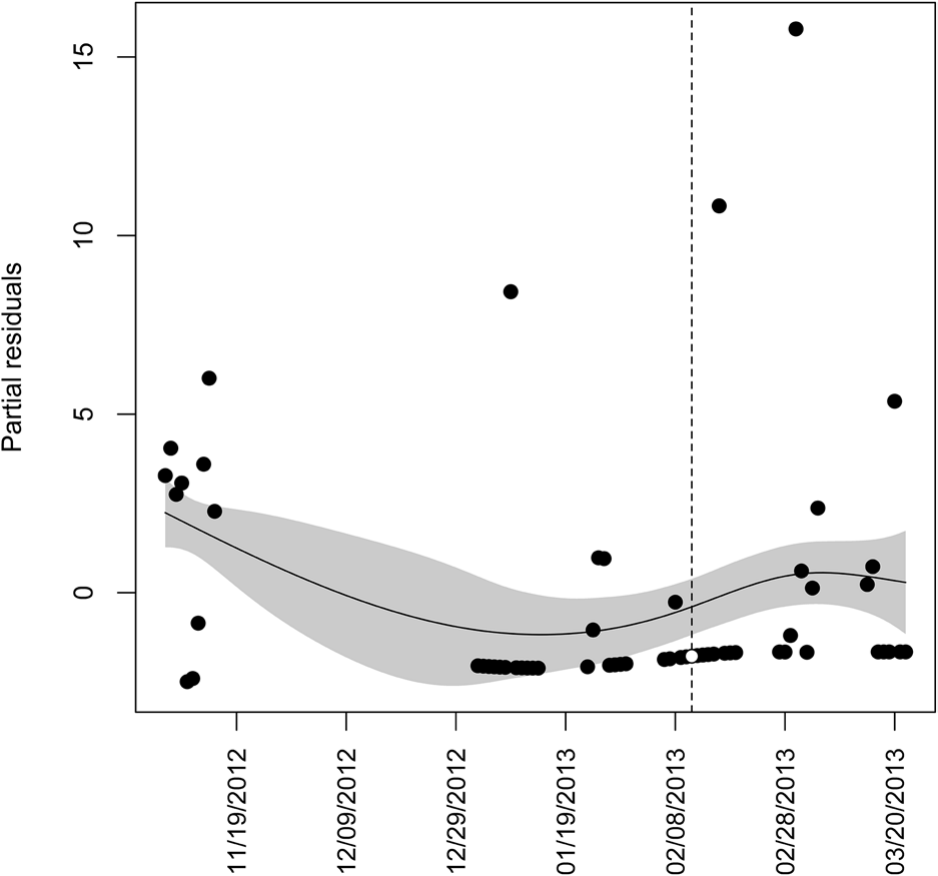
Example figure of partial residuals of the number of acoustic files per night by date for cave C40 in winter in southeastern Idaho in which there was no response of Townsend’s big-eared bats to researchers entering caves. Points are partial residuals for each acoustic-survey date. The dashed vertical line and white circle represent the residual from the night following when we entered the cave to conduct a survey. Error envelopes represent ± 2 *SEs*.

To document the effect of researchers entering caves on cave-exiting flight of bats, we first used generalized additive models (GAMs)^55,56^. Separate models were created for Townsend’s big-eared bats and western small-footed myotis. We used Poisson or quasipoisson error terms with log link functions. The latter error terms were used when Poisson models were over dispersed. The response variable was the number of acoustic files per night^48,53^. We produced GAM thin-plate splines using default settings in the R package mgcv^57,58^, with the number of acoustic files per night as a smoothed function of date, for each of nine surveyed caves, and for each surveyed year. We then extracted model residuals. Those values were the working residuals from the iteratively reweighted least squares (IRLS) models. Those residuals were weighted by the square root of the IRLS weights, so that residuals had constant variance if the specified model was correct^58^. We then determined if partial residuals resulting from the GAM models were > 2 *SE*s of the model fit, indicating higher than expected bat activity (Fig. 1).

We then used logistic regression to determine the variables that influenced cave-exiting flight of bats. Thus, our binary response-variable outcomes were either (1) indicating that the partial residual of bat cave-exiting flight was > 2 *SE*s of the model fit, or (0) indicating that the partial residual of bat cave-exiting flight was ˂ 2 *SE*s of the model fit (Fig. 1). Covariates of the logistic regression model included a derived researcher disturbance factor (i.e., number of researchers × total time in a cave in minutes) and cave length. The following variables were recorded on the day of our hibernacula surveys: number of hibernating bats , number of clusters of bats, and cluster size of bats in each cave. We also included mean ambient temperature (°C), maximum minus minimum barometric pressure (hPa) over night, and mean wind speed (m/s) as those variables have the greatest effect on natural cave-exiting behavior of bats in winter in our study area^22^. Weather data were collected from the closest (within 20 km) National Oceanic and Atmospheric Administration weather station to our study caves every 5 min. from ½ hour before sunset to ½ hour after sunrise each day^22^.

We tested for autocorrelated predictors using Pearson’s correlation and determined that cave length, number of hibernating bats in years surveys were conducted, number of clusters of bats, and cluster size of bats were strongly correlated with our derived researcher disturbance factor (*p* ≤ 0.05). Thus, we excluded the former variables and only included researcher disturbance factor, mean temperature, barometric pressure, and mean wind speed in our models. Barometric pressure resulted in rank-deficient models that would not converge and therefore was removed as a covariate. Previous work by our group indicated that temperature and wind speed were more important predictors of normal bat cave-exiting behavior than barometric pressure^22^. We created a saturated model using all three explanatory variables (researcher disturbance factor, temperature, and wind speed). We used AIC model selection to find the optimal approximating model (minimum AIC) with respect to the saturated model^59,60^. We assessed the fit of the logistic regression model using the Area Under Curve (AUC) value, which is a measure of model classification efficacy^61^. AUC values > 0.8 denote a good model, while AUC values > 0.9 indicate an excellent model. We used the R statistical environment version 4.2.2 for all analyses^62^, with relieance on the packages mgcv and MASS.

## Results

Acoustic detectors set at 9 caves recorded 8953 files (Townsend’s big-eared bat = 2227 files and western small-footed myotis = 6726 files) over 815 detector nights. Mean (± s.d.) number of files recorded per night across caves during winter for Townsend’s big-eared bats was 3 (s.d. = 11.2 files, range = 0 to 220 files) and 8 (s.d. = 32.1 files, range = 0 to 570 files) for western small-footed myotis. We conducted 17 hibernacula surveys in 9 caves (Table 1). The mean date of surveys was February 12 (s.d. = 17.6 days). We counted a mean of 102 (s.d. = 150.7) Townsend’s big-eared bats and 4 (s.d. = 7.9) western small-footed myotis per cave during surveys (Table 1). On nights following those 17 hibernacula surveys, detectors recorded 166 files (Townsend’s big-eared bat = 101 files and western small-footed myotis = 65 files). Mean number of files recorded per night across caves the night following hibernacula surveys (*n* = 17) for Townsend’s big-eared bats was 6 (s.d. = 16.0 files, range = 0 to 63 files) and for western small-footed myotis was = 4 (s.d. = 10.0 files, range = 0 to 41 files).

**Figure 2:**
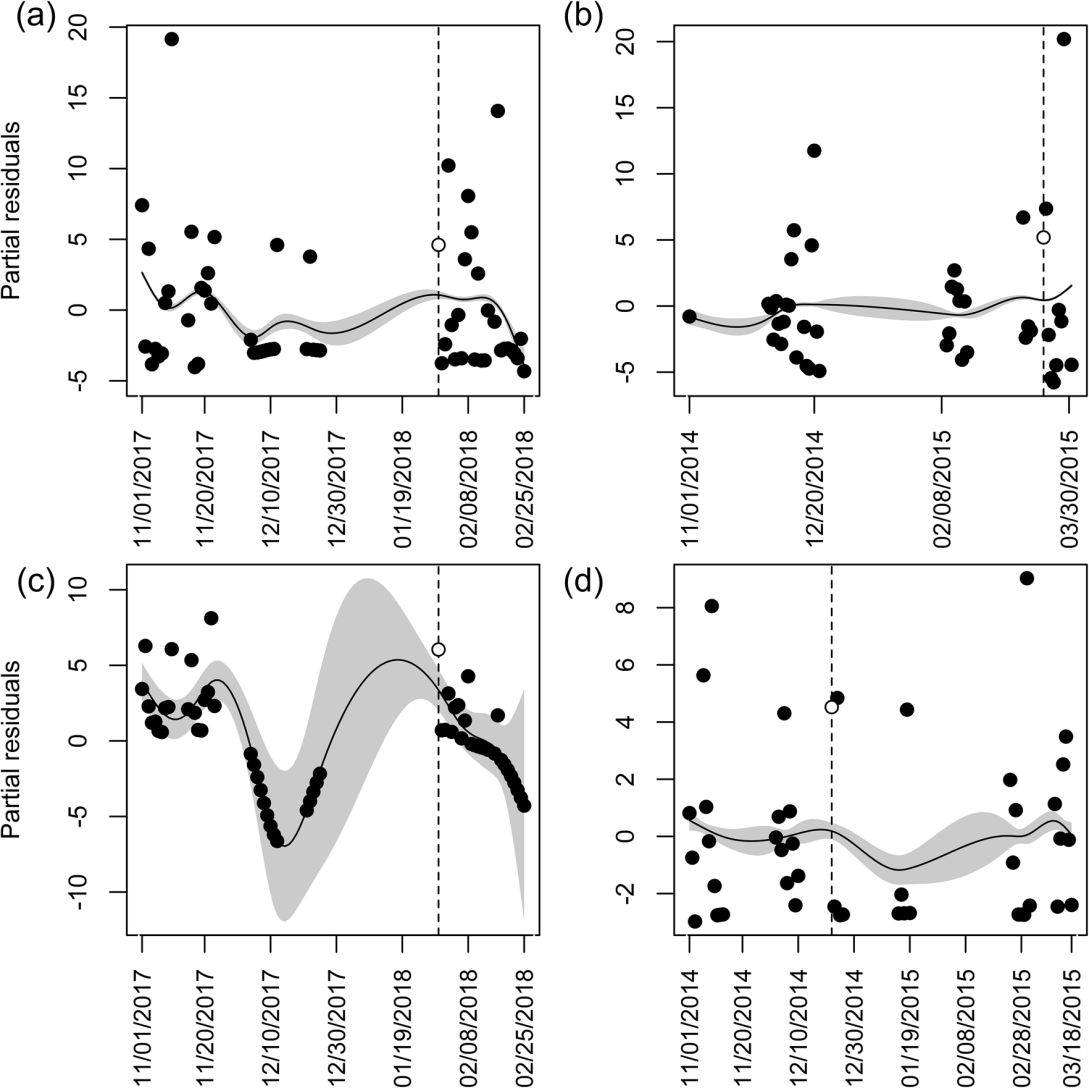
Partial residuals of the number of acoustic files per night by date for caves in which there was a response of bats to researchers entering caves to conduct hibernacula surveys in southeastern Idaho; (**a**) western small-footed myotis in cave C40, (**b**, **c**) Townsend’s big-eared bats in cave C40, and (**d**) Townsend’s big-eared bats in cave C54. Points are partial residuals for each date. The dashed vertical line and white circle represent the residual from the night following when we entered the cave to conduct a survey. Error envelopes represent ± 2 s.e.m.

Townsend’s big-eared bats and western small-footed myotis did not fly out of caves more than predicted during 82% of the nights (*n* = 14) following our surveys. While conducting hibernacula surveys, we documented one Townsend’s big-eared bat and one unidentified bat flying in cave C40 in January 2018. We did not record bats flying during any other survey. We only noted a response from western small-footed myotis to researchers entering cave C40 in January 2018 (Fig. 2a), which was the cave and year with the highest disturbance factor (Table 1). Townsend’s big-eared bats also responded to researchers entering that cave in that year (Fig. 2c). We also documented a response from Townsend’s big-eared bats to researchers entering cave C40 in 2014/2015 (Fig. 2b) and in 2017/2018 (Fig. 2c) and in cave C54 in 2014/2015 (Fig. 2d), and thus used logistic regression to identify covariates that influenced bats responding to researchers in caves C40 and C54 in those two years. Our top ranked model included disturbance factor and temperature [logit(bat cave-exiting flight) = -3.817 + 0.013 × Disturbance factor + 0.472 × Temperature]. However, in our optimal approximating model, temperature was not significant (χ^2^ = 2.75, *d.f.* = 1, *p* = 0.097), and only the disturbance factor we derived was statistically important (χ^2^ = 4.99, *d.f.* = 1, *p* = 0.026). Additionally, disturbance factor was in the top four models (Table 2).

**Table 2:** Model results describing disturbance of hibernating Townsend’s big-eared bats the night following 17 hibernacula surveys we conducted in 9 caves from 2013 to 2018 in southeastern Idaho, USA. We report model structure, number of estimated parameters (K), rankings (AIC and ΔAIC), and model weights.

Model	K	AIC	ΔAIC	Akaike weight
Disturbance + Temperature	3	11.9	0	0.33
Disturbance	2	12.7	0.75	0.23
Disturbance + Wind	3	13.6	1.69	0.14
Disturbance + Temperature + Wind	4	13.6	1.73	0.14
Temperature	2	14.9	2.99	0.07
Temperature + Wind	3	15.6	3.66	0.05
Wind	2	17.9	6.04	0.02
Intercept only model	1	27.9	16	0.02

**Figure 3:**
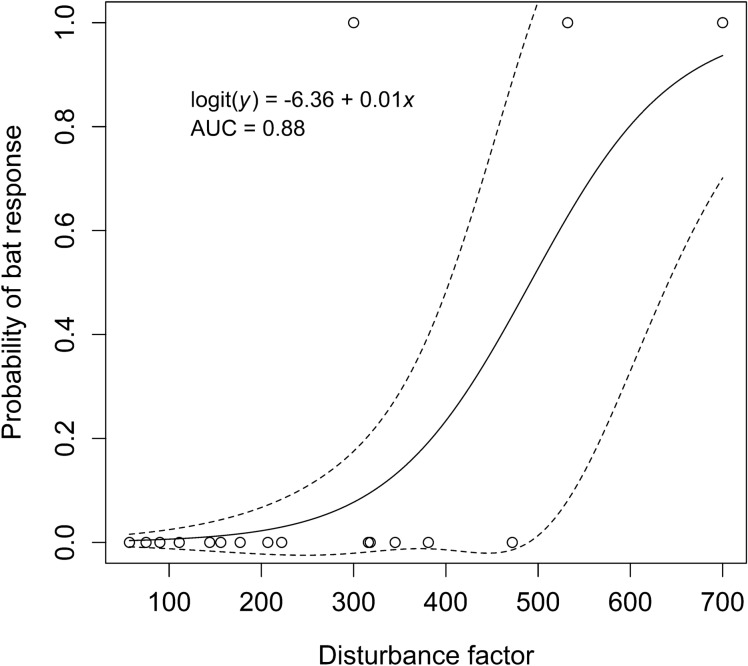
Relationship between probability of Townsend’s big-eared bats flying out of caves in response to the number of researchers × total time a cave (i.e., a derived researcher disturbance factor) in southeastern Idaho from November to March from 2013 to 2018, when holding temperature constant at its mean. Dashed lines represent ± 1 s.e.m. of the fitted value.

The mean disturbance factor value when Townsend’s big-eared bats responded to researchers entering the two caves (cave C40 in 2014/2015 and 2017/2018 and cave C54 in 2014/2015) to conduct hibernacula surveys was 511 (s.d. = 200.9, range = 300 to 700; Table 1); whereas the mean disturbance factor value when bats did not respond to researchers entering caves to conduct hibernacula surveys was 219 (s.d. = 127.7, range = 57 to 472; Table 1). In the case of this species, the probability of bats responding to researchers in a cave increased as disturbance factor increased (Fig. 3), while holding temperature constant. For example, when the disturbance factor was 500 there was a probability of 0.53 of causing bats to arouse from torpor, fly, and exit the cave the night following a survey when holding air temperature at its mean (-5.4 °C, Fig. 3). Additionally, there was a probability of approximately 0.94 of bats arousing from torpor, flying, and exiting the cave when the disturbance factor is 700 (Fig. 3).

## Discussion

We predicted that the number of acoustic files recorded the night following hibernacula surveys would be greater than during nights following no hibernacula surveys for Townsend’s big-eared bats and western small-footed myotis. Our results indicated that the study species did not exit caves more than predicted during 82% of the nights following surveys. In a study using a passive acoustic recorder, researchers did not document an increase in flight when conducting counts of hibernating bats ranging from 48 to 67 individuals across two years when one researcher entered the cave up to five times a month for a mean of 50 min^43^. Researchers have recommended that knowledge gained from entering caves and censusing bats during hibernation may outweigh potential disturbance due to human visitation if such information can be used to understand and conserve bat species^31,63^. Our results indicate that in our study area, most surveys caused no response from bats flying out of caves, and that, for example, a researcher disturbance factor value of ≤ 300 (e.g., 3 researchers in a cave for ≤ 100 min.) would produce a < 10% probability of bats arousing from torpor and flying out of a cave when holding temperature constant (Fig. 3). Importantly, other species in other areas may be more sensitive to such disturbance.

The prediction that the number of files recorded the night following hibernacula surveys would be greater than during nights following no surveys was upheld during two winters in two caves for Townsend’s big-eared bats. Those cave/year combinations had two of the highest disturbance factors. That outcome occurred even though the intensity of echolocation calls of Townsend’s big-eared bats is low compared with calls of western small-footed myotis^64,65^. In our study area, Townsend’s big-eared bats often occupy open areas and hang from the ceiling of a cave during hibernation^7,34,44^. Therefore, this species is often susceptible to disturbance in hibernacula, especially repeated disturbance^1,66^.

Previous work indicates that only a small proportion of bats in a population may be sensitive to nontactile disturbance during hibernacula surveys^38^, and that the response to disturbance can differ by individual^37^. We documented a significant positive relationship between the probability of Townsend’s big-eared bats flying out of caves and a derived researcher disturbance factor. Researchers spending more time in a cave increased the probability of bats arousing and flying out of a cave the night following surveys. Townsend’s big-eared bats occupy over 2,500 caves and mines across western North America^26^. Our logistic regression model for predicting the probability of cave-exiting flights of Townsend’s big-eared bats can be used to help design hibernacula surveys for this species. Specifically, the model can be used in other areas to estimate the probability of disturbing this species based on the number of researchers entering caves and mines, as well as how long those researchers spend conducting hibernacula surveys.

We only documented western small-footed myotis responding to researchers entering one cave during one survey in 2018, although that species was recorded flying out of caves naturally on nights following no surveys three times more so than Townsend’s big-eared bats. That cave (C40) in that year had the highest disturbance factor (700) of any hibernacula survey in our study. Furthermore, that cave had a disturbance-factor value that was three times higher than the mean disturbance factor for all other caves when bats did not respond to researchers entering caves. In our study caves during hibernation, western small-footed myotis are not as prevalent as Townsend’s big-eared bats^6,7,44^. Moreover, during hibernacula surveys, we often observed western small-footed myotis wedged up to 25 cm in cracks of the walls and ceilings. We hypothesize that western small-footed myotis were not as likely to be disturbed by researchers and arouse from torpor compared with Townsend’s big-eared bats, because of the cracks-in-the-wall roosting behavior of this myotis species. Thus, roosting behavior of species should be considered when documenting responses of bats to researchers conducting hibernacula surveys.

Our surveys were conducted on one day a year in each cave, and our detectors were placed outside of cave entrances. In other studies documenting a response of bats to researchers entering one mine^37,38^, eight artificial bunkers^33^, and one cave^43^, researchers entered those hibernacula from a minimum of six to a maximum of 25 times in a winter. As we only entered caves one day in a year, our results indicate that even when researchers only enter hibernacula once a winter, those entries can still cause bats to arouse and fly out of caves. Moreover, Stapelfeldt, et al.^33^, Thomas^38^, and Park et al.^43^ also set acoustic recording devices up to 20 m inside of the structures they monitored, which differed from our set-up of acoustic devices outside of caves. Although we did not detect bats flying outside of most caves the night following hibernacula surveys, bats still could have aroused and flown inside the cave, and our acoustic detectors would not have detected them flying. Therefore, our acoustic detectors likely underestimated bats flying in hibernacula. Future research should test the correlation between bats flying inside the cave with those flying outside the cave when disturbed by researchers entering caves to conduct hibernacula surveys, especially in large caves.

White-nose syndrome and other threats have negatively affected bat populations^67–69^. These threats make it essential to enter caves and monitor long-term population trends of these mammals^6,29,70^. Management strategies limiting researchers entering caves need to balance between costs of disturbances to bats and benefits of knowledge gained for the conservation of these mammals^31,63^, however, such strategies should be evaluated on a site-specific basis. Our results indicate that researchers entering caves in a hibernation season did not cause bats to arouse and fly out of caves more than predicted during the night following 82% of surveys. Our logistic regression model did indicate a relationship between the number of researchers and time spent in a cave with an increasing probability of Townsend’s big-eared bats arousing from torpor, flying, and exiting a cave following a hibernacula survey. Our results allow for the prediction of the probability of disturbing hibernating Townsend’s big-eared bats based on the group size of researchers and the amount of time researchers spend in caves. For example, if 5 researchers are in one cave for 100 min (i.e., a disturbance factor was 500) there is a 53% probability of causing bats to arouse from torpor and fly out of the cave. This information can help biologists plan for how many researchers and how long those researchers are in a cave to minimize disturbing bats.

## Data Availability

The datasets generated during and/or analysed during the current study are available from the corresponding author on reasonable request.
